# Eating More and Fighting Less: Social Foraging Is a Potential Advantage for Successful Expansion of Bird Source Populations

**DOI:** 10.3390/biology11101496

**Published:** 2022-10-12

**Authors:** Xiang Li, Xiaochen Wang, Jinyao Lu, Li Li, Dongming Li, Xiaoying Xing, Fumin Lei

**Affiliations:** 1 College of Wildlife and Protected Area, Northeast Forestry University, Harbin 150040, China; 2 Northeast Asia Biodiversity Research Center, Harbin 150040, China; 3 Hunan Biodiversity Conservation Center, Changsha 410004, China; 4 Key Laboratory of Animal Physiology, Biochemistry and Molecular Biology of Hebei Province, College of Life Sciences, Hebei Normal University, Shijiazhuang 050024, China; 5 Key Laboratory of Zoological Systematics and Evolution, Institute of Zoology, Chinese Academy of Sciences, Beijing 100101, China; 6 University of Chinese Academy of Sciences, Beijing 100101, China

**Keywords:** distribution change, *Pycnonotus sinensis*, social foraging, expansion advantage, source population, competition, expansion potential

## Abstract

**Simple Summary:**

Understanding why some animal species expand their distributions but others do not under the same global climatic fluctuations and habitat changes is essential to determine expansion mechanisms and predict alien species invasion. However, whether the source population of an expansive species has the potential to survive in a new environment after expansion is rarely evaluated. Social foraging is considered beneficial in obtaining food and increasing population establishment. However, competition will increase under the social context of group foraging. In this laboratory research, whether social foraging could promote individuals to consume more novel food in an unfamiliar environment, which can facilitate survival and population establishment, was tested in a source population of a successful expansive bird, the Light-vented Bulbul (*Pycnonotus sinensis*). Compared with the sympatric, nonexpansive relative species Collared Finchbill (*Spizixos semitorques*), bulbuls had longer latencies to consume novel food, but they increased eating times when transferred from solitary to group status and had higher possibilities of eating with their companions. In addition, more than two individuals eating together is significantly more frequent in bulbuls than finchbills. Therefore, social foraging can be a critical species-specific trait that is a potential advantage for expansion because it weakens competition among individuals. This study provides testable hypotheses to explain mechanisms of successful expansions and to predict alien species expansion and possibilities for control.

**Abstract:**

Animals can expand distributions in response to climatic and environmental changes, but the potential expansive ability of a source population is rarely evaluated using designed experiments. Group foraging can increase survival in new environments, but it also increases intraspecific competition. The trade-off between benefit and conflict needs to be determined. The expanding Light-vented Bulbul *Pycnonotus sinensis* was used as a model to test mechanisms promoting successful expansion. Social foraging and its advantages were evaluated using lab-designed feeding trials. Consuming novel foods was compared between bulbuls and a sympatric, nonexpansive relative species, the finchbill *Spizixos semitorques*, from native areas at both solitary and social levels. Bulbuls increased their eating times when transferred from solitary to group, whereas social context did not affect finchbills. Bulbuls were significantly more likely to eat with their companions than finchbills when in a group. Thus, exploring food resources in a bulbul source population was facilitated by social context, indicating that social foraging is an important means by which birds successfully expand and respond to environmental changes. This research increases understanding of successful expansion mechanisms and will consequently help predict invasive potentials of alien species.

## 1. Introduction

How animals respond to global climate changes and habitat transformation is currently a hot topic in ecology, evolutionary biology, and conservation biology. Expanding to new areas is one of the most important mechanisms to respond to environmental changes [1], but not all species can colonize and establish breeding populations in new environments. Some species expand successfully, whereas the distributions of others shrink or populations decrease or even become endangered, such as the recently classified as critically endangered species of Yellow-breasted Bunting [2]. Therefore, species-specific life-history strategies are most likely key factors driving successful expansion. Several mechanisms drive successful expansion, including behavioral, physiological, morphological, and life-history traits [3,4,5,6,7]. Exploration, in which an animal acts to initiate a change in its environment [8], or responding to a stimulus [9], may be related to ecological factors [10] or cognition factors [11]. Exploring and utilizing novel food resources are associated with successful expansion and are two of the most important abilities needed to survive in new circumstances after expansion [7,12,13,14]. It has been found that trying new foods may explain how House Sparrows (*Passer domesticus*) have so successfully invaded new areas [12]. In some other cases, expansive birds have higher foraging efficiency [13], but the underlying mechanisms to explain increases in foraging efficiency remain unclear.

Social foraging is a critical mechanism by which populations successfully establish in new habitats after expansion, although it is associated with both benefits and negative effects [15,16]. Social foraging species can be better colonizers than solitary species because social foraging can increase the probability of locating food, exploring new food sources, and detecting predators [17,18]. The invasive House Sparrow with familiar companions will land sooner for feeding, which demonstrates that social environment influences colonizers’ behavioral responses [15]. However, such hypotheses have not been validated by strong empirical support [13,19]. Group foraging can increase fitness by obtaining valuable information quickly to increase foraging efficiency, by saving energy spent on vigilance, and by reducing predation risk because of the dilutive effect of a group [15,16]. Such benefits should be particularly useful for an expansive species when confronted with a novel environment, because the ability to rapidly gather and share information about food resources and enemies may determine survival and increase foraging efficiency [20,21,22]. However, social foraging can result in fierce intraspecific competition among group members for food [19,23]. Nevertheless, sociality is characteristic of some expansive taxa, and it is commonly considered to promote successful colonization, although only a few studies examine the connection between successful expansion and sociality [19,20]. Investigating the tradeoffs for range-expansion species between the benefits of joint foraging and the negative effects of competition under social foraging would help us discover new mechanisms of successful expansion.

Although assessment of whether a species gains potential advantages in exploring novel food resources before expansion is lacking, it would be critical for predicting potential invaders. Most related studies focus on the differences between invasive and native populations, or the differences between frontier and core invasive populations [24,25,26,27]. However, few studies examine the differences between expansive species and sympatric nonexpansive closely related species in a native area, which could determine whether expansive species exhibit advanced behavioral traits before expansion that would be useful for living in new environments [28].

Although there are 66 Pycnonotidae birds which reside in southern China [29], there are few records of expansion in other bulbuls [30]. Only the Light-vented Bulbul *Pycnonotus sinensis* (hereafter, bulbuls) has expanded its distribution naturally from the 1930s in China and has established stable breeding populations in the north over the last 40 years [31,32]. To date, it has become a dominant local bird species in some northern areas and is observed as frequently as is the Tree Sparrow *Passer montanus* [33]. Successful colonization indicates bulbuls have high adaptability and survival ability in novel environments. Collared Finchbill *Spizixos semitorques* (hereafter, finchbills), a closely related species of bulbuls, also established breeding populations northward in similar habitats and even became resident in the Palearctic realm, although there are no reports of further expansion [34,35]. Both bulbuls and finchbills share similar life-history traits, such as both being typical omnivores that consume a broad range of foods including fruit, vegetables, seeds, insects, and human discards [36]. Diet generalism is an important and well-known predictor of successful invasions [37,38] which indicates both the bulbul and finchbill have potential for expansion ability when feeding habits are considered. There was previous research that has demonstrated that bulbuls have vocal learning ability and showed innovation in breeding song after expansion to the north [31], but whether bulbuls explore more rapidly and more easily accept novel foods than finchbills when given the choice has not been tested. Furthermore, bulbuls and finchbills exhibit different group size in the nonbreeding season when in the wild, i.e., bulbuls forage and fly in groups of dozens of individuals, whereas finchbills are often observed singly or in groups of a few individuals. Overall, group size is much smaller with finchbills than with bulbuls [36]. Therefore, it was hypothesized that social foraging is important in determining bulbul survival, especially in new environments after expansion. Considering that their native distribution ranges occur sympatrically in southern China, bulbuls and finchbills are excellent model species for investigating the tradeoffs between benefits and competition under group foraging that promotes successful expansion.

In the present study, to investigate whether the bulbul and finchbill exhibit different potential ability to consume novel foods and how the social context affects those choices, behavioral tests were combined with captive feeding trials to examine novel food selections by bulbuls and finchbills under both solitary and social contexts in the source populations. The specific objectives were the following: (1) quantify the willingness of bulbuls and the sympatric relative finchbills in a solitary context to taste novel foods in an unfamiliar environment; (2) evaluate whether exploring novel foods changed when birds were moved from solitary to social context and whether there was a difference in response between species; and (3) investigate whether bulbuls and finchbills obtained more food in a social than solitary context because of, for example, weak competition among individuals.

## 2. Materials and Methods

### 2.1. Subjects and Husbandry

Eighteen Light-vented Bulbuls and eight Collared Finchbills were caught using mist nests at Changsha (Hunan, China, 112°58′ E, 28°11′ N) in 2018 with scientific sampling permission from the Hunan Forestry Administration Department. All 26 birds were transported to the Hunan Wildlife Rescue and Rehabilitation Center and participated in experiments 1 and 2. Individuals were marked with colored plastic leg bands. Birds were then released into outdoor roofed group aviaries (3 m × 3 m × 4 m = height × length × width) where they could fly and move freely during a 10-d habituation period to captivity before testing. Food and water were always available, and birds were fed common fruits, vegetables, and commercial *Tenebrio molitor* according to their natural diets in the wild. Birds could bask in sunshine and bathe with clear water daily to keep feathers clean, similar to life in the wild.

In the novel food experiments, birds were held in small individual cages (0.6 m × 0.4 m × 0.4 m) indoors, and cages were covered with white paper to block visual signals before novel food experiments. Individual cages were equipped with a perch and small boxes with food and water. This arrangement facilitated bird adaptation to individual housing.

### 2.2. Procedures

#### 2.2.1. Novel Food Preparation

Both bulbul and finchbill are frugivorous, in addition to catching insects during the breeding season. Therefore, five types of fresh fruits that were not planted locally were selected as the novel foods used in experiments: (a) cherry *Cerasus pseudocerasus*, (b) water chestnut *Eleocharis dulcis*, (c) dragon fruit *Hylocereus undatus*, (d) Chinese flowering quince *Chaenomeles sinensis*, and (e) mango *Mangifera indica*. Multiple types of fruits were used to exclude bias caused by different food types and low number of individual birds. Foods were cut into small pieces with the same size and shape except for the cherries (which were presented whole), and were put into the cages, and the foods were visually identical in size and shape and novel to each captive bird. Most bulbuls and finchbills in the wild would be unlikely to have previous experience with the fruits.

Experiments were conducted early in the morning following overnight food deprivation. Both bulbuls and finchbills were tested on the same day and at the same time, but experiments of the same fruit type were carried out in different rooms to ensure presenting it to both species at the same pace. To avoid a neophobic response caused by food location, novel foods were placed in the same location used for daily feeding of the bulbuls and finchbills. The order in which individual birds experienced each novel food was randomized in order to minimize order effects [39,40].

#### 2.2.2. Experiment 1: Solitary Foraging of Novel Food

In experiment 1, how bulbuls and finchbills explored novel foods was evaluated and whether there was species-specific variation in exploration propensity under the same unfamiliar environment in the laboratory was determined. To measure how bulbuls and finchbills accepted novel foods, the 18 bulbuls and 8 finchbills were transferred to individual cages and left alone for 30 min before being presented with a novel food. According to observations before the experiment of birds living freely in the outdoor aviaries, both bulbuls and finchbills would eat fresh fruits and live worms within 20 min, at the most. Thus, 30 min was set as the observation time during experiments to evaluate how birds consumed novel foods. A video camera recorded the entire 30-min process following food introduction to determine whether and how birds accessed novel foods.

#### 2.2.3. Experiment 2: Group Foraging and Competition for Novel Food

According to field observations, bulbuls always forage and move in groups of several to dozens of individuals in the nonbreeding season, whereas finchbills seldom group into large flocks. The purpose of experiment 2 was to investigate whether the propensity to explore novel foods changed, and how birds competed for food, when in a group compared with being solitary. Four individual birds were tested together in the group context for bulbuls and finchbills in an unfamiliar experimental cage inside a new room. In addition, interspecies differences between bulbuls and finchbills were determined. Whether individuals fought for food under starvation in a social context was also assessed. The “fight for food” was defined as two birds pecking or chasing one another from food using the bill.

### 2.3. Behavioral Measurements

In experiments 1 and 2, several behavioral variables were measured from video recordings to quantify birds’ propensity to explore and accept novel food resources under solitary and group contexts [12]: time to start eating a novel food (t1), number of times eating novel food (N), and percentage of time spent consuming novel food during 30 min (DP).

To measure competition or social compatibility when offered novel food in a group, number of times and time duration of “fight for food” (F and FD, respectively) and number of times and time duration of more than two individuals consuming food together (C and CD, respectively) were measured within 30 min. 

### 2.4. Statistical Analyses

To investigate variations in propensity to explore novel foods between bulbuls and finchbills observed in experiment 1, *t*-tests were used in analyzing number of times and time duration of more than two individuals consuming food together (C and CD, respectively), and also time duration of “fight for food” (FD), chi-square tests were used in comparing changes from solitary to group contexts, and Mann–Whitney U tests were used in analyzing times of “fight for food” (F). All data were tested for normality by one-sample Kolmogorov–Smirnov tests before *t*-tests were performed, and alpha levels were set at 0.05. All statistical analyses were conducted using IBM SPSS Statistics for Windows, v21.0 (IBM Corp., Armonk, NY, USA).

The influence of sex, species, social context, and novel food type on the number of times eating novel food (N), time to start eating a novel food (t1), and percentage of time spent consuming novel food during 30 min (DP) were investigated. These potential effects were analyzed using Generalized Linear Mixed Models (GLMMs), which are often used when data are non-normally distributed and random effects possibly account for part of the variance. All data were analyzed using R v4.0.5 [41]. All statistical tests were two-tailed. The significance threshold was set at α = 0.05. The models were fitted with three dependent variables (N, t1, DP) using the ‘glmer’ function within the package ‘lme4’ (1.1-29) for R v4.0.5. Each dependent variable was analyzed using a separate model [42]: time eating novel food (N), time to start eating a novel food (t1), and percentage of time spent consuming novel food during 30 min (DP). Sex, species, novel food type, context (repeated measure, alone, i.e., solitary, and group), and their interaction were fitted as categorical fixed effects [15]. The dependent variables relative to each individual were (i) foraging times (N, times); (ii) time to start eating novel food (t1, seconds); and (iii) percentage of time eating during 30 min (DP, percentage, while duration itself was recorded as seconds). The three dependent variables were modeled with gamma distribution (log link).

Estimates and significance of fixed effects were obtained using the ‘Anova’ function within the ‘car’ package, whereas the ‘confint.merMod’ function within the ‘lme4’ package was used to obtain confidence intervals. The identity of the bird was treated as a random effect.

## 3. Results

### 3.1. Variations in Foraging Behavior among Solitary and Group Bulbuls and Finchbills

**Figure 1:**
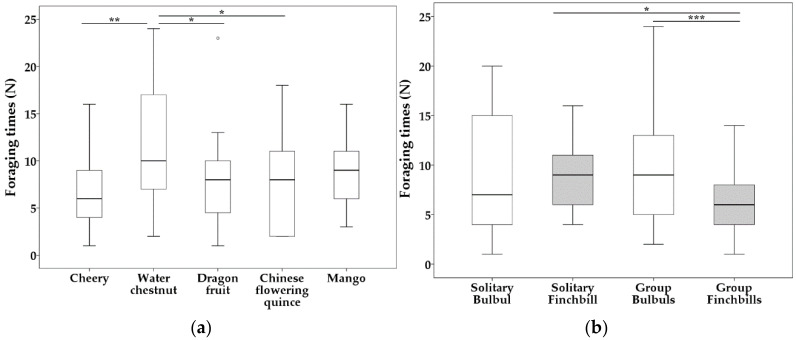
Boxplots of influence of novel food type (**a**) and species × social context (**b**) on foraging times (N) for Ligth-vented Bulbul (white bars) and Collared Finchbill (grey bars). Bars show the maximum observed value (upper part) and the minimum observed value (lower part). Line and asterisk(s) above the boxplots indicate statistically significant comparisons between the two groups. Upper edge of the box shows the upper quartiles (Q3), while the lower edge of the box shows the lower quartiles (Q1). The middle line in the box shows the median. Abnormal values are shown as small circles (for outliers) above the boxplots but under the line. *** *p* < 0.001. ** *p* < 0.01. * *p* < 0.05.

The main effect ‘novel food type’ had a significant influence on foraging times (N) (*df* = 4, *F* = 3.043, *p* = 0.021). Novel food water chestnut (type b) was foraged significantly more times than other novel food types (Table 1, Figure 1). The species × social context interaction was also significant (*df* = 1, *F* = 7.333, *p* = 0.008). Collared Finchbills foraged significantly more times when they were solitary, compared to group Collared Finchbills (Table 1, Figure 1). While in group, Light-vented Bulbuls foraged more times than Collared Finchbills (Table 1, Figure 1). However, the main effects ‘sex’ and ‘social context’ were not significant (sex: *df* = 1, *F* = 1.772, *p* = 0.187; social context: *df* = 1, *F* = 1.699, *p* = 0.196).

**Figure 2:**
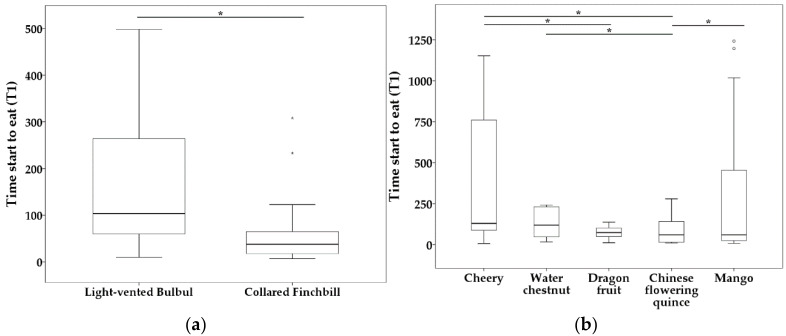
Boxplots of influence of species (**a**) and novel food type (**b**) on time to start eating novel food (t1). Bars show the maximum observed value (upper part) and the minimum observed value (lower part). Line and asterisk(s) above the boxplots indicate statistically significant comparisons between the two groups. Upper edge of the box shows the upper quartiles (Q3), while the lower edge of the box shows the lower quartiles (Q1). The middle line in the box shows the median. Abnormal values are shown as small circles for outliers and asterisk for extrema above the boxplots but under the line. * *p* < 0.05.

The main effects ‘species’ and ‘novel food type’ had a significant influence on time to start eating novel food (t1) (species: *df* = 1, *F* = 6.366, *p* = 0.013; novel food type: *df* = 4, *F* = 5.547, *p* = 0.001;). Light-vented Bulbuls start to eat novel food later than Collared Finchbills (Table 2, Figure 2). Novel food cherry (type a) was eaten later than both dragon fruit (type c) and Chinese flowering quince (type d), and water chestnut (type b) was also eaten later than Chinese flowering quince (type d). However, Chinese flowering quince (type d) was eaten sooner than mango (type e) (Table 2, Figure 2). However, the sex × social context and species × social context interactions were not significant (sex × social context: *df* = 1, *F* = 1.981, *p* = 0.163; species × social context: *df* = 1, *F* = 2.399, *p* = 0.125). Likewise, the main effects ‘sex’ and ‘social context’ were also not significant (sex: *df* = 1, *F* = 0.277, *p* = 0.600; social context: *df*= 1, *F* = 1.583, *p* = 0.212).

**Figure 3:**
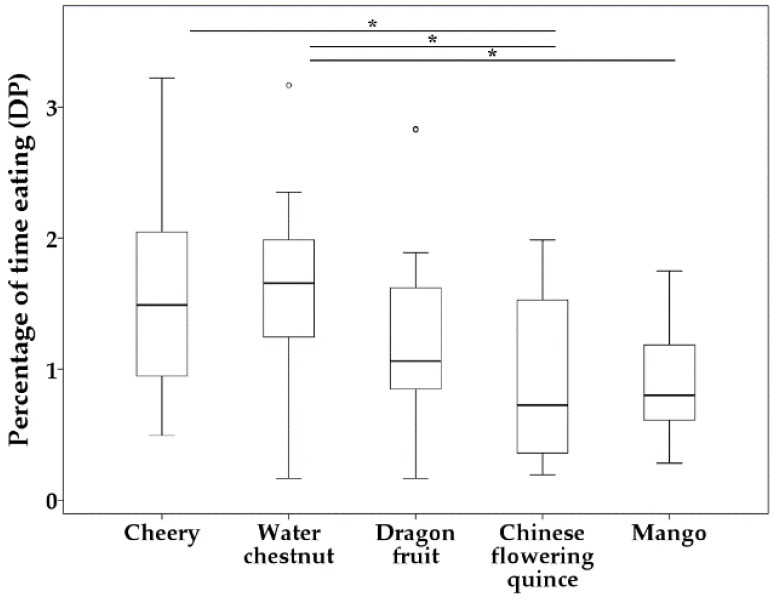
Boxplots of influence of novel food type on percentage of time eating during 30 min (DP). Bars show the maximum observed value (upper part) and the minimum observed value (lower part). Line and asterisk(s) above the boxplots indicate statistically significant comparisons between the two groups. Upper edge of the box shows the upper quartiles (Q3), while the lower edge of the box shows the lower quartiles (Q1). The middle line in the box shows the median. Abnormal values are shown as small circles for outliers above the boxplots but under the line. * *p* < 0.05.

The main effect ‘novel food type’ had a significant influence on percentage of time eating during 30 min (DP) (*df* = 4, *F* = 2.944, *p* = 0.025). Novel food Chinese flowering quince (type d) was eaten for shorter time duration than both cherry (type a) and water chestnut (type b) during 30 min (Table 3), while water chestnut (type b) was eaten for longer time duration than mango (type e) during 30 min (Table 3, Figure 3). However, the main effects ‘sex’, ‘species’, and ‘social context’ were not significant (sex: *df* = 1, *F* = 0.244, *p* = 0.623; species: *df* = 1, *F* = 0.095, *p* = 0.759; social context: *df* = 1, *F* = 2.048, *p* = 0.156). Also, the sex × social context and species × social context interactions were not significant (sex × social context: *df* = 1, *F* = 0.157, *p* = 0.693; species × social context: *df* = 1, *F* = 0.127, *p* = 0.722).

### 3.2. Comparison of Foraging Behavior between Bulbuls and Finchbills

When individual birds were presented novel foods, 50% of bulbuls (9 of 18) and 100% of finchbills (8 of 8) ate the novel food provided, and thus, finchbills were significantly more likely to consume novel food resources (Chi-square test, χ^2^ = 3.846, *df* = 1, *p* = 0.050). 

### 3.3. Changes from Solitary to Group Context

Compared with solitary foraging, bulbuls in a group significantly increased their time to start eating novel food (t1, χ^2^ = 4.832, *df* = 1, *p* = 0.032) and also time spent consuming novel food (DP, 0.645 ± 0.280, *p* = 0.024). By contrast, when in a group, finchbills significantly decreased the time spent consuming novel food (DP, 0.724 ± 0.349, *p* = 0.041). However, the start time to touch novel food was not affected (t1, χ^2^ = 0.116, *df* = 1, *p* = 0.733; Figure 1).

### 3.4. Intraspecific Food Competition

**Figure 4:**
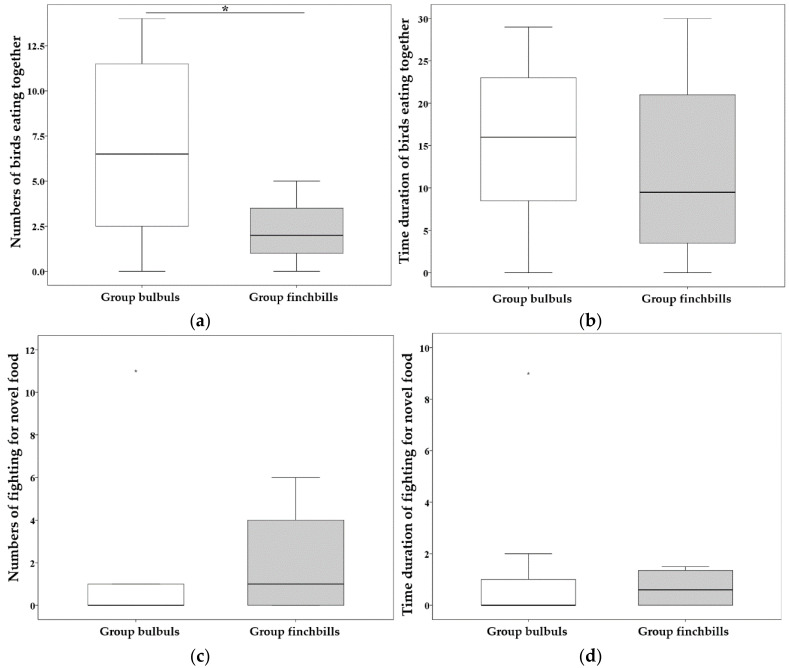
Boxplots of shared novel foods by more than two birds and fights for novel food between Light-vented Bulbuls (*n* = 4) and Collared Finchbills (*n* = 4) when foraging socially (G). (**a**) Number of times more than two birds ate novel food together (C), line and asterisk(s) above the boxplots indicate statistically significant differences between the two groups.; (**b**) time duration of more than two birds eating novel food together (CD); (**c**) number of fights for novel food (F); and (**d**) time duration of fights for novel food (FD). Bars show the maximum observed value (upper part) and the minimum observed value (lower part). Upper edge of the box shows the upper quartiles (Q3), while the lower edge of the box shows the lower quartiles (Q1). The middle line in the box shows the median. Abnormal values are shown as asterisks for extrema above the boxplots but under the line.

In social context, a total of 8 individuals (4 bulbuls and 4 finchbills) and 20 samples (16 for bulbuls and 4 for finchbills) were recorded showing behaviors of eating together or fighting for food. When social, bulbuls had significantly more times with more than two individuals eating novel food together (C, *t* = 2.829, *df* = 18, *p* = 0.015; Figure 4). However, time duration of eating novel food together (CT, *t* = 0.640, *p* = 0.530; Figure 4), number of fights for novel food (F, *t* = −0.736, *p* = 0.462; Figure 4), and time duration of fights for novel food (FT, *t* = 0.336, *p* = 0.741; Figure 4) were not significant in social context.

## 4. Discussion

Compared with solitary bulbul foraging, when in a group bulbuls spent significantly more time consuming novel food, whereas finchbills ate novel foods fewer times. Bulbuls showed greater social compatibility than finchbills, and more than two individuals ate novel food together significantly more often. In addition, bulbuls had fewer and shorter fights for novel food than those of finchbills, though not significantly.

### 4.1. Finchbills Foraged More Novel Food in a Solitary Than Social Context

It is essential that, when establishing populations after colonization, invasive species be good at searching and consuming novel food resources in new environments [12,43]. With wide niche breadth, generalists can consume a broad range of foods and survive better in novel environmental conditions than more specialized species [7,44,45]. The bulbuls ate the novel foods provided in the experiments in this study, which could explain the successful expansion of bulbuls on the basis of consuming novel food resources. Other bird species also use novel foods, and expansive birds can even choose high-nutrition foods in new environments. The invasive Common Myna *Sturnus tristis* has a clear preference for high-protein foods [7].

However, consuming novel foods alone cannot explain the successful expansion of bulbuls, because both bulbuls and finchbills ate the novel foods provided in the experiment, which might be explained by the omnivory of the birds in the wild. In this study, all finchbills ate the novel foods, whereas only half of bulbuls ate novel foods. Therefore, because finchbills are more likely to use novel food resources, access to novel resources may not be the only factor explaining why bulbuls succeed in surviving after expansion into new environments. Bulbuls even had significantly longer latencies to eat the novel foods, and the solitary finchbill was more likely to consume novel food within shorter times in the experiment, so the willingness to explore novel foods is likely not the primary advantageous trait for bulbuls to survive in new environments.

### 4.2. Bulbuls Consumed More Novel Food after Transferring from Solitary to Group Foraging

Compared with solitary foraging, the presence of a conspecific can facilitate [46,47,48] or delay [16] the acceptance of novel food. To obtain more food, birds can observe and learn from one another which foods can be safely eaten. Social foraging can include benefits such as obtaining information on food localities [23] and learning new foraging behaviors, as occurred in the classic research when tits acquired a new skill to harvest food by opening a milk bottle cover to obtain nutritious lipids [49].

In this study, when transferred from solitary to group status, the social bulbuls benefited by being in a group. The social context promoted bulbul consumption of novel foods, whereas it had no effects on finchbills. First, all finchbills ate the novel foods in solitary and social context. By contrast, only half of individual bulbuls in a solitary context ate novel foods, but all individual bulbuls under a social context ate novel foods. Increased consumption of novel food under social context may be caused by learning from or following decisions by other individuals. Second, compared with finchbills, social bulbuls ate significantly more times. The result suggests that social context changed trends and ways of exploring novel foods for bulbuls, which may occur because bulbuls prefer to observe others exploring and touching novel foods first. The faster ravens delay approaching novel objects until observing the response of a companion [50]. Therefore, it was hypothesized that bulbul foraging strategy and behaviors were based on observing responses of other individuals to novel foods. For finchbills, the time to start eating novel foods after being transferred from solitary to group foraging was similar. The result indicates that an individual finchbill is much more independent than an individual bulbul and that the social context did not affect their trends and speed of consuming novel food.

Therefore, social context promoted bulbul consumption of more novel food resources in a new environment, which could be one of the important drivers and advantages in expansion. This conclusion is consistent with field observations of bulbuls always foraging and participating in activities in groups of up to dozens of individuals in the nonbreeding season. By contrast, finchbills are often observed foraging and participating in activities singly or in relatively small groups with only a few individuals [36]. Such differences support the idea that bulbuls in a group have increased exploring ability and are therefore more likely to discover and consume novel food resources. Similarly, the invasive Red-billed Leiothrix *Leiothrix lutea* is more competitive than local birds because of higher feeding efficiency in new distribution areas of Europe [51].

### 4.3. Bulbuls Shared More but Competed Less in Social Context

In social context, competition among individuals in a group can delay or decrease access to food resources [16]. Therefore, the tradeoffs between benefits and negative effects when an animal enters a group need to be investigated. One mechanism to maintain group stability or increase benefits is to decrease competition, and the results in the current study indicated that this mechanism was operating. Compared with finchbills, more than two individual bulbuls shared food more times, and bulbuls also had fewer and shorter fights, though not significantly. Thus, individual bulbuls were more compatible than individual finchbills, which were more competitive. Therefore, the results support the speculation that more compatible or weak competition is the key mechanism to guarantee or improve benefits when under social context [17].

Although there was fighting among bulbuls, the competition was relatively weak in a bulbul group and did not decrease access to food, in contrast to social finchbills. Decreased competition and increased sharing of food resources are critical for bulbuls when exploring and eating novel foods. When a few birds find and consume a novel food in a new environment, other birds quickly obtain the information by learning from or mimicking the pioneers and they will also eat the novel food. Thus, group behavior can increase the survival success of bulbul populations in new environments.

## 5. Conclusions

When consumption of novel food resources was compared between the successful expansive bulbul and the sporadically expansive finchbill, bulbuls consumed more food when in a group than as individuals, and weak competition within bulbul groups might be the core mechanism that increased group feeding efficiency. By contrast, finchbills in groups consumed less novel food and were not affected by a change from solitary to social context. Obtaining information about food by observing or learning from other individuals might influence bulbuls’ foraging strategy. The study showed that a social context was critical for bulbuls to adapt by finding novel food resources. Thus, the expansive potential of bulbuls might be explained by decreasing competition or increasing compatibility among individuals in a group, and by learning from or mimicking other individuals to learn how to obtain more food and increase survival.

## Figures and Tables

**Table 1:** Effects of fixed factors on foraging times (N) for solitary and group Light-vented Bulbuls and Collared Finchbills.

Fixed Effect	Comparison	Estimate	5%CI	95%CI	*p* Value
Sex	Male vs. Female	1.648	−0.751	4.047	0.176
Species	Light-vented Bulbul vs. Collared Finchbill	0.807	−1.632	3.246	0.512
Social Context	Solitary vs. Group	1.718	−1.060	4.496	0.222
Novel Food Type	a vs. b	−4.478	−7.582	−1.374	**0.005**
	a vs. c	−0.405	−2.895	2.085	0.747
	a vs. d	−1.008	−3.508	1.491	0.425
	a vs. e	−2.338	−4.992	0.317	0.084
	b vs. c	4.076	0.890	7.263	**0.013**
	b vs. d	3.459	0.277	6.642	**0.033**
	b vs. e	1.892	−1.407	5.191	0.257
	c vs. d	−0.603	−3.226	2.020	0.649
	c vs. e	−1.933	−4.660	0.795	0.162
	d vs. e	−1.330	−4.055	1.396	0.335
Sex × Social Context	Male: Solitary vs. Group	2.049	−1.576	5.673	0.264
	Female: Solitary vs. Group	1.434	−2.468	5.335	0.467
Social Context × Sex	Solitary: Male vs. Female	1.980	−2.884	6.843	0.421
	Group: Male vs. Female	1.365	−0.484	3.214	0.146
Species × Social Context	Light-vented Bulbul: Solitary vs. Group	−1.784	−5.038	1.469	0.279
	Collared Finchbill: Solitary vs. Group	4.938	0.561	9.316	**0.028**
Social Context × Species	Solitary: Light-vented Bulbul vs. Collared Finchbill	−2.928	−7.844	1.989	0.240
	Group: Light-vented Bulbul vs. Collared Finchbill	3.795	1.817	5.773	**<0.001**
Random Effect		**variance**	**±SE**		
Individual Identity	□	0.325	0.050	□	□

Coefficients and 95% confidence intervals (CI) are presented; statistically significant comparisons (zero is not included in the interval) are in bold. *p*-values were obtained with Tukey’s method adjusted for multiple comparisons. “Individual identity” was fitted as a random effect, with associated variance shown. Food type: (a) cherry, (b) water chestnut, (c) dragon fruit, (d) Chinese flowering quince, and (e) mango.

**Table 2:** Effects of fixed factors on time to start eating novel food (t1) for solitary and group Light-vented Bulbuls and Collared Finchbills.

Fixed Effect	Comparison	Estimate	5%CI	95%CI	*p* Value
Sex	Male vs. Female	−33.644	−165.862	98.573	0.614
Species	Light-vented Bulbul vs. Collared Finchbill	166.747	17.030	316.465	**0.029**
Social Context	Solitary vs. Group	−86.333	−213.983	41.318	0.182
Novel Food Type	a vs. b	142.194	−131.106	415.494	0.304
	a vs. c	291.266	45.710	536.823	**0.013**
	a vs. d	313.216	67.853	558.579	**0.013**
	a vs. e	140.774	−130.333	411.882	0.305
	b vs. c	149.072	−2.524	300.669	0.054
	b vs. d	171.022	23.214	318.831	**0.024**
	b vs. e	−1.420	−193.027	190.188	0.988
	c vs. d	0.547	−50.153	94.053	0.547
	c vs. e	−150.492	−302.676	1.692	0.053
	d vs. e	−172.442	−322.695	−22.188	**0.026**
Sex × Social Context	Male: Solitary vs. Group	4.094	−136.622	144.810	0.954
	Female: Solitary vs. Group	−203.603	−434.561	27.354	0.083
Social Context × Sex	Solitary: Male vs. Female	43.602	−128.504	215.709	0.616
	Group: Male vs. Female	−164.095	−355.588	27.398	0.092
Species × Social Context	Light-vented Bulbul: Solitary vs. Group	24.470	−266.387	315.326	0.868
	Collared Finchbill: Solitary vs. Group	−120.031	−262.203	22.141	0.097
Social Context × Species	Solitary: Light-vented Bulbul vs. Collared Finchbill	222.697	−55.667	501.062	0.115
	Group: Light-vented Bulbul vs. Collared Finchbill	78.197	−74.240	230.635	0.311
Random Effect		**variance**	**±SE**		
Individual Identity	□	1.729	0.265	□	□

Coefficients and 95% confidence intervals (CI) are presented; statistically significant comparisons (zero is not included in the interval) are in bold. *p*-values were obtained with Tukey’s method adjusted for multiple comparisons. “Individual identity” was fitted as a random effect, with associated variance shown. Food type: (a) cherry, (b) water chestnut, (c) dragon fruit, (d) Chinese flowering quince, and (e) mango.

**Table 3:** Effects of fixed factors on percentage of time eating during 30 min (DP) for solitary and group Light-vented Bulbuls and Collared Finchbills.

Fixed Effect	Comparison	Estimate	5%CI	95%CI	*p* Value
Sex	Male vs. Female	−0.124	−0.633	0.384	0.628
Species	Light-vented Bulbul vs. Collared Finchbill	0.077	−0.578	0.424	0.760
Social Context	Solitary vs. Group	0.385	−0.188	0.958	0.185
Novel Food Type	a vs. b	−0.104	−0.792	0.583	0.763
	a vs. c	0.192	−0.489	0.873	0.576
	a vs. d	0.660	0.075	1.225	**0.027**
	a vs. e	0.555	−0.015	1.126	0.056
	b vs. c	0.297	−0.379	0.973	0.385
	b vs. d	0.754	0.186	1.323	**0.010**
	b vs. e	0.660	0.095	1.224	**0.022**
	c vs. d	0.458	−0.110	1.025	0.113
	c vs. e	0.363	−0.197	0.923	0.201
	d vs. e	−0.095	−0.513	0.324	0.655
Sex × Social Context	Male: Solitary vs. Group	0.465	−0.189	1.119	0.161
	Female: Solitary vs. Group	0.297	−0.628	1.221	0.525
Social Context × Sex	Solitary: Male vs. Female	−0.028	−1.063	1.007	0.957
	Group: Male vs. Female	−0.196	−0.596	0.204	0.332
Species × Social Context	Light-vented Bulbul: Solitary vs. Group	0.285	−0.433	1.003	0.432
	Collared Finchbill: Solitary vs. Group	0.490	−0.375	1.356	0.263
Social Context × Species	Solitary: Light-vented Bulbul vs. Collared Finchbill	−0.193	−1.191	0.805	0.702
	Group: Light-vented Bulbul vs. Collared Finchbill	0.012	−0.431	0.455	0.956
Random Effect		**variance**	**±SE**		
Individual Identity	□	0.358	0.055	□	□

Coefficients and 95% confidence intervals (CI) are presented; statistically significant comparisons (zero is not included in the interval) are in bold. *p*-values were obtained with Tukey’s method adjusted for multiple comparisons. “Individual identity” was fitted as a random effect, with associated variance shown. Food type: (a) cherry, (b) water chestnut, (c) dragon fruit, (d) Chinese flowering quince, and (e) mango.

## Data Availability

Publicly available datasets were analyzed in this study. This data can be found here: [https://github.com/ravenli0719/raven-s-walnut.git] (accessed on 15 August 2022).
